# Antibacterial, Antibiofilm, and Antioxidant Activities of Aqueous Crude *Gymnema inodorum* Leaf Extract against Vancomycin-Resistant *Enterococcus faecium*

**DOI:** 10.3390/microorganisms12071399

**Published:** 2024-07-11

**Authors:** Sakaewan Ounjaijean, Voravuth Somsak, Morteza Saki, Watcharapong Mitsuwan, Chonticha Romyasamit

**Affiliations:** 1 Research Institute for Health Sciences, Chiang Mai University, Chiang Mai 50200, Thailand; 2 School of Allied Health Sciences, Walailak University, Nakhon Si Thammarat 80160, Thailand; 3 Research Excellence Center for Innovation and Health Products, Walailak University, Nakhon Si Thammarat 80160, Thailand; 4 Department of Microbiology, Faculty of Medicine, Ahvaz Jundishapur University of Medical Sciences, Ahvaz, Iran; mortezasaki1981@gmail.com; 5 Akkhraratchakumari Veterinary College, Walailak University, Nakhon Si Thammarat 80160, Thailand; watcharapong.mi@wu.ac.th; 6 Center of Excellence in Innovation of Essential Oil and Bioactive Compounds, Walailak University, Nakhon Si Thammarat 80160, Thailand

**Keywords:** *Gymnema inodorum*, plant extract, vancomycin-resistant *Enterococci*, antibacterial compound, bacterial resistance

## Abstract

Vancomycin-resistant *Enterococcus faecium* (VREF) causes nosocomial infections with high mortality and morbidity rates. This study aimed to evaluate the antibacterial and antibiofilm activities of aqueous crude *Gymnema inodorum* leaf extract (GIE) against the VREF ATCC 700221 strain. The antimicrobial activity of GIE against VREF was performed using disk diffusion and broth microdilution. The antibiofilm activities were evaluated using the crystal violet staining assay. The antioxidant potential was evaluated. Preliminary screening of the antimicrobial activity of 50 and 100 µg/disk of GIE against VREF revealed inhibition zones of 8.33 ± 0.58 mm and 8.67 ± 0.29 mm, respectively. Additionally, the minimum inhibitory concentration (MIC) and minimum bactericidal concentration (MBC) values against VREF were 125 and ≥ 250 mg/mL, respectively. SEM analysis showed that treatment with GIE caused morphological changes, including incomplete cell division, damaged cell walls, and cell content leakage, suggesting a disruption of bacterial cells. GIE also inhibited and eradicated biofilms formed by VREF. The extract exhibited antioxidant activities in the DPPH and ABTS assays. While GIE shows potential as an antibacterial and antibiofilm agent, further studies are necessary to fully understand the underlying mechanisms and optimize its use for therapeutic applications.

## 1. Introduction

*Enterococcus faecium* is a Gram-positive coccus occurring in pairs or chains [1]. The pathogen originates from the gastrointestinal tract. It can then spread and cause severe hospital-associated infections in healthcare settings worldwide, such as urinary tract infections, wound infections, intra-abdominal and pelvic region infections, and bloodstream infections [2,3]. Therapy is complicated by resistance to multiple antibiotics. *E. faecium* generally harbors a range of intrinsic and acquired resistance genes, such as glycopeptides (vancomycin and teicoplanin), beta-lactams (ampicillin and penicillin), aminoglycosides (gentamicin or streptomycin), and macrolides [3,4]. In addition, the widespread emergence of vancomycin-resistant enterococci (VRE) has caused further concern due to the high mortality rate [5]. The World Health Organization published a list of the most common bacteria associated with nosocomial infections in 2017, with VRE being ranked as the third most common cause of nosocomial infections worldwide [6].

Regarding pathogenesis, *E. faecium* possesses a wide range of virulence factors, such as collagen-binding adhesin of *E. faecium* (Acm), cytolysin (CylA), enterococcal surface protein (Esp), gelatinase (GelE), *vanA*, and *vanB* mobile gene clusters [7,8]. *E. faecium’s* capsule structures are the primary virulence factors involved in promoting biofilm formation and evading neutrophil killing [2,9]. Due to the property of biofilm formation, *E. faecium* becomes aggregated and difficult to eradicate. This mechanism allows pathogenic bacteria to resist higher antibiotic concentrations, resulting in chronic infections and antibiotic-resistant strains [2]. Although antibiotics are effective against bacteria, treating biofilm infections requires high concentrations of antibiotics, generally above peak serum levels. Consequently, latent and recurring infection therapies are less successful.

Therefore, alternative therapeutics such as natural products, especially plant extracts, have been used for several years as the basis for treatment and have attracted widespread interest [10,11]. Previous studies have demonstrated that plant extracts such as *Epilobium angustifolium* L. [12], *Gymnema sylvestre* [13], Japanese traditional (Kampo) medicine [11], and *Commiphora pedunculata* exhibit antimicrobial activity against *E. faecium* [14]. In this study, we focused on *Gymnema inodorum* (Lour.) Decne., which is an endemic plant species of Southeast Asia, including Southern China, Indonesia, the Philippines, and Thailand. This plant belongs to the family Asclepiadaceae [15,16], and its leaves contain several phytochemical compounds, including phenolics, flavonoids, terpenoids, and glycosides. Methanol extract provides the highest phenolic content, while ethanol extract provides the highest flavonoid content [17]. *G. inodorum* has been used in herbal medicine to treat diseases such as diabetes mellitus, rheumatoid arthritis, and gout [15]. Moreover, phytochemical compounds, known for their antioxidant properties, can mitigate oxidative stress linked to bacterial infections. These natural compounds help neutralize harmful free radicals, reducing damage during infections. Additionally, some phytochemicals have direct antibacterial effects, offering potential for new treatments [18,19]. Previous studies have highlighted the antimicrobial activities of related species, such as *Gymnema sylvestre*, which has shown inhibitory effects against various Gram-positive and Gram-negative bacteria, including *S. aureus* and *E. coli* [13,20]. Similarly, *Gymnema lactiferum* has demonstrated potential antimicrobial activities against pathogenic bacteria [21]. Moreover, *G. inodorum* extract showed potent antimalarial activity against *Plasmodium berghei* infection [16,22,23]. However, to the best of our knowledge, there are no reports on the antibacterial activity of *G. Inodorum* against vancomycin-resistant *E. faecium* (VREF). Therefore, this study aimed to evaluate the antibacterial and antibiofilm activities of aqueous crude *Gymnema inodorum* leaf extract (GIE) against the VREF ATCC 700221 strain, in addition to its antioxidant property.

## 2. Materials and Methods

### 2.1. Bacterial Strain and Growth Conditions

*E. faecium* ATCC 700221, containing *vanA* resistance genes confirmed by polymerase chain reaction (PCR) and resistant to vancomycin, was kindly provided by Miss Phanvasri Saengsuwan, Department of Biomedical Sciences and Biomedical Engineering, Faculty of Medicine, Prince of Songkla University, Hatyai, Songkhla, Thailand. *E. faecium* ATCC 700221 was cultured on tryptic soy agar (TSA) obtained from HiMedia (Mumbai, India) and incubated at 37 °C for 18 h. Then, the bacterial cultures were inoculated into tryptic soy broth (TSB) (HiMedia, Mumbai, India) and then incubated at 37 °C for 18 h. The cultures were preserved in TSB containing 20% glycerol at −80 °C until needed for further experiments.

### 2.2. Preparation of Plant Extracts

Leaves of *G. inodorum* were kindly provided by Dr. Sakaewan Ounjaijean at Chiang Mai University. GIE was prepared according to the previously described method with slight modifications [16]. Briefly, a 100 g sample of powdered leaves was extracted with 500 mL of distilled water (DW) at 60 °C for 6 h with occasional stirring. The extract was then filtered through Whatman No. 1 filter paper to remove any particulate matter. The filtrate was concentrated under reduced pressure using a rotary evaporator at 40 °C until a thick, crude extract was obtained. The dried powdered form of aqueous crude GIE was dissolved in DW.

### 2.3. Gas Chromatography–Mass Spectrometry (GC–MS) Analysis

Phytochemicals in *G. inodorum* extract were detected using GC–MS analysis using Agilent Technologies 7890 B (GC) equipped with a 5977A Mass Selective Detector (MS). Briefly, the analysis utilized a VF-WAXms capillary column (Agilent Technologies, Santa Clara, CA, USA) measuring 30 m × 0.25 mm, with a film thickness of 0.25 µm. Helium served as the carrier gas at a flow rate of 1 mL/min. The column temperature was initially set at 60 °C, increased to 160 °C at a rate of 10 °C per minute, and then ramped up to 250 °C at a rate of 2.5 °C per min, with a hold time of 15 min. Mass spectrometry was performed in the electron ionization mode at 70 eV, with a source temperature of 230 °C, scanning continuously from 35 to 500 *m*/*z*. The phytochemicals in the *G. inodorum* leaf extract were identified by comparing their mass spectral data with entries in the Wiley library.

### 2.4. Antibiotic Susceptibility of VREF

The antibiotic susceptibility profile of *E. faecium* ATCC 700221 was evaluated using the antibiotic sensitivity test according to the methods described in the Clinical and Laboratory Standards Institute (CLSI) 2021 guidelines [24]. The reference strain was suspended and inoculated onto Mueller–Hinton agar (MHA) (HiMedia, Mumbai, India). Antibiotic disks (Oxoid, Hampshire, UK) containing ampicillin (AMP; 10 µg), vancomycin (VA; 30 µg), erythromycin (E; 15 µg), tetracycline (TE; 15 µg), and clindamycin (DA; 2 µg) were placed on culture plates. The plates were incubated at 37 °C for 24 h. The zone of inhibition was measured with calipers according to CLSI 2021 guidelines.

### 2.5. Evaluation of the Antibacterial Effects of GIE

#### 2.5.1. Disk Diffusion Assay

The antibacterial activity of GIE *against* VREF was evaluated using the disk diffusion method according to Arun et al. (2014) [20]. Briefly, 10 µL of plant extract was applied to sterile filter paper disks (6 mm diameter) to yield final contents of 100 and 50 mg per disk. Overnight cultures of *E. faecium* ATCC 700221 were suspended in Mueller–Hinton agar broth (MHB) (HiMedia, Mumbai, India) to a cell density of 1 × 10^8^ CFU/mL and spread on MHA plates. The disks were placed on pathogen-inoculated agar plates and incubated at 37 °C for 18 h. Antimicrobial activity was evaluated by measuring the zone of inhibition against the test organisms in triplicate. Deionized water was the negative control, and TE was the positive control.

#### 2.5.2. Determination of Minimum Inhibitory Concentration (MIC) and Minimum Bactericidal Concentration (MBC)

The MIC and MBC of GIE against VREF were evaluated according to methods described in the CLSI 2021 guidelines [24]. Briefly, GIE was serially diluted in a 96-well microtiter plate to a final concentration of 250 to 0.98 mg/mL in MHB. A suspension of *E. faecium* (5 × 10^5^ CFU/mL) was added to each well, and plates were incubated at 37 °C for 18 h. Tetracycline (TE) and deionized water (DI) were the positive and negative controls, respectively. To determine MIC values, 0.05% resazurin (Thermo Fisher Scientific, Lancashire, UK) was added. The MIC was defined as the lowest concentration that completely inhibited bacterial growth, indicated by a blue color [24]. MBC values were determined by streaking cultures from wells with significant MIC results onto TSA plates to assess bacterial viability.

### 2.6. Morphology Study of VREF by Scanning Electron Microscopy (SEM)

The effects of GIE on *E. faecium* ATCC 700221 morphology were examined using SEM following the method by Kulnanan et al. (2021) [25] with slight modification. Both treated and untreated *E. faecium* ATCC 700221 cells were fixed in 2.5% (*v*/*v*) glutaraldehyde (Sigma-Aldrich, St. Louis, MI, USA) in 0.1 M phosphate buffer for 24 h at 4 °C. The samples were then dehydrated through a graded ethanol series (20%, 50%, 70%, 90%, and 100%) for 15 min each, followed by two dehydration steps in 100% ethanol for 15 min. The samples were air-dried at room temperature for 30 min, mounted on stubs, and coated with gold for 3 min. The bacterial morphology after treatment with the extract was observed using a field emission scanning electron microscope (Oxford Instruments, Quanta, Japan).

### 2.7. Biofilm-Forming Ability of E. faecium ATCC 700221

The biofilm-forming ability of *E. faecium* ATCC 700221 was determined by the crystal violet assay [25].

### 2.8. Biofilm Inhibition Assay

Antibiofilm properties of GIE were evaluated using the crystal violet assay, following the method of Sornsenee et al. (2021) with slight modifications [26]. Briefly, overnight cultures of *E. faecium* ATCC 700221 were suspended in MHB to a cell density of 5 × 10^5^ CFU/mL and then inoculated into 96-well plates supplemented with 1×, 2×, 4×, and 8× MIC of GIE. DI served as the negative control. The plates were incubated at 37 °C for 24 h. After incubation, the medium was removed, and the biofilms were washed three times with phosphate-buffered saline (PBS, pH 7.4) and fixed with 200 µL of methanol for 15 min. The biofilm was then stained with 200 µL of crystal violet solution (0.1% *w*/*v*) for 15 min. Excess dye was removed by rinsing the wells four times with DI. The stained biofilms were dissolved in 99% ethanol, and absorbance was measured at 570 nm. The relative percentage of biofilm inhibition was calculated using the following formula:

[(OD_570 nm_ without extract − OD_570 nm_ with extract)/OD_570 nm_ without extract]  ×  100.

### 2.9. Biofilm Eradication Assay

The biofilm eradication effects of GIE on the established biofilm of *E. faecium* ATCC 700221 were evaluated as described by Sornsenee et al. (2021) [26] with slight modification. Briefly, the overnight culture of *E. faecium* ATCC 700221 was suspended in MHB to a cell density of 5 × 10^5^ CFU/mL, inoculated into 96-well plates, and then incubated at 37 °C for 2 days. Then, GIE (1×, 2×, 4×, and 8× MIC of GIE) was added to eradicate the established biofilms and incubated at 37 °C for 24 h. Next, the plates were removed, gently washed three times with PBS, and stained with 200 uL of crystal violet solution. DI was the negative control. The percentage of biofilm eradication was calculated using the following equation:

Biofilm eradication (%) = [(OD_570 nm_ without extract − OD_570 nm_ with extract)/OD_570 nm_ without extract]  ×  100.

### 2.10. Determination of Antioxidant Activity

#### 2.10.1. Total Phenolic Content (TPC) Assay

The Folin–Ciocalteu method was widely used to measure the total polyphenolic content in plant extract. It was used to indirectly determine the total amounts of the amino acids tyrosine and tryptophan with improved sensitivity and good reproducibility, as described by Zongo et al. (2010) [27] with some modifications. GIE was diluted in DI to a concentration of 1 mg/mL. Then, 100 µL of 0.1 M sodium carbonate (Na_2_CO_3_) solution and 100 µL of 10% Folin–Ciocalteu reagent (Sigma-Aldrich, St. Louis, MI, USA) were mixed in the well of a 96-well plate and incubated for 30 min at room temperature in the dark. After incubation, the absorbance was measured at 750 nm. A standard curve was generated using gallic acid with a 1.569–200 µg/mL concentration range. The total polyphenolic content (TPC) was expressed as milligrams of gallic acid equivalents (GAE) per gram of dry GIE.

#### 2.10.2. Free Radical Scavenging Assay

This is one of the known mechanisms by which antioxidants inhibit lipid oxidation. Radical scavengers can directly interact with peroxide radicals and scavenge them in order to stop the peroxidation chain events and enhance the potency and stability of plant extract. 2,2-Diphenyl-1-picrylhydrazyl (DPPH) (CID: 2735032) and 2,2′-Azinobis (3-ethylbenzothiazoline-6-sulfonic acid) (CID: 9570474) scavenging methods have been used to evaluate the antioxidant activity of compounds due to the simple, rapid, sensitive, and reproducible procedures.

- 2,2-Diphenyl-1-picrylhydrazyl (DPPH) radical scavenging activity

The free radical scavenging activities of GIE were assessed using the DPPH assay with Trolox (Sigma-Aldrich, St. Louis, MI, USA) as the standard. This assay followed the procedure described by Dunkhunthod et al. (2021) with slight modifications [28]. An amount of 100 μg/mL of GIE or 1.56–100 μg/mL ascorbic acid standard in absolute methanol was mixed with 180 μL of DPPH reagent in a 96-well plate. The reaction mixture was kept in the dark for 30 min, and the absorbance was measured at 517 nm using a microplate reader. The scavenging ability was calculated as scavenging activity (%) = 100 × [Abs of control − (Abs of sample − Abs of blank)]/Abs of control.

- 2,2′-Azinobis (3-ethylbenzothiazoline-6-sulfonic acid) (ABTS) diammonium salt radical scavenging activity

The ABTS radical scavenging activity of GIE was evaluated using an ABTS decolorization assay as described by Sornsenee et al. (2021) [26]. The percent inhibition of absorbance at 734 nm was calculated using the following equation:

% Scavenging activity = 100 × (Abs of control − (Abs of sample − Abs of blank))/Abs of control.

### 2.11. Statistical Analysis

Experiments were conducted in triplicate. Data are expressed as mean ± standard error. Statistical analysis was conducted using the two-tailed unpaired Student’s *t*-test. In all analyses, a *p*-value < 0.05 was considered to be statistically significant. GraphPad Prism version 9 software (GraphPad Software, La Jolla, CA, USA) was used for all analyses.

## 3. Results

### 3.1. Identification of the Phytochemicals in Gymnema inodorum Extract Using GC–MS Analysis

**Figure 1:**
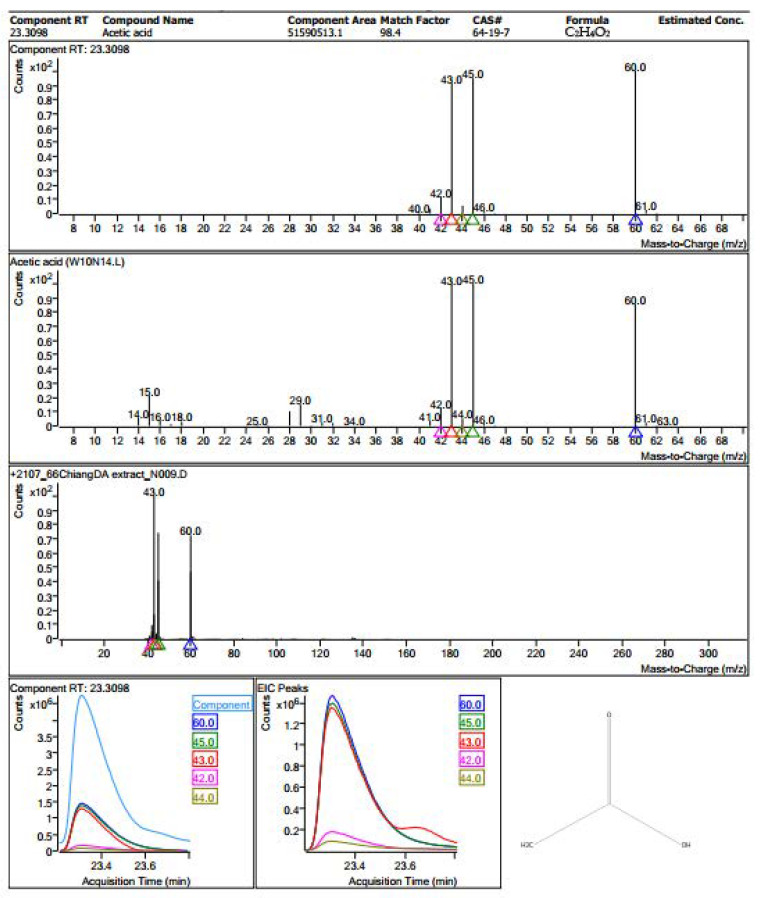
GC–MS spectrum and phytochemical structures of acetic acid presented in *Gymnema inodorum* extract.

The phytochemicals in the extract were identified by GC–MS analysis. A total of 53 compounds were detected from *Gymnema inodorum* extract (Table 1 and Figures S1–S5). Acetic acid was the major phytochemical presented in the extract (Figure 1), followed by 2-Pyrrolidinone, 1,2,3-Propanetriol, and Pyrrolo[1,2-a] pyrazine-1,4-dione, hexahydro-3-(2-methylpropyl), respectively (Figures S1–S5). The result reveals that 12.8% total peak area of acetic acid was found in *Gymnema inodorum* extract.

### 3.2. Antibiotic Susceptibility and Biofilm Formation of VREF

*E. faecium* ATCC 700221 was susceptible to ampicillin and tetracycline but resistant to erythromycin, clindamycin, and vancomycin. This strain showed strong biofilm-forming ability.

### 3.3. Antibacterial Activity of GIE

The preliminary screening of the antimicrobial activity of 50 and 100 µg/disk of GIE against *E. faecium* ATCC 700221 revealed inhibition zones of 8.33 ± 0.58 mm and 8.67 ± 0.29 mm, respectively. The TE zone inhibition as a control positive was equal to 25 ± 0.00 mm. Additionally, the MIC and MBC of the GIE were 125 mg/mL and ≥250 mg/mL, respectively (Table 2).

### 3.4. Effect of GIE on VREF Morphology

**Figure 2:**
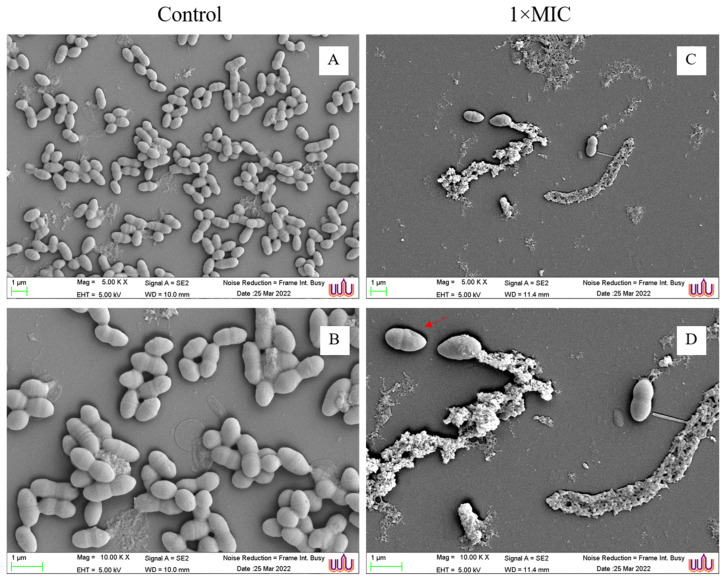
Morphology of *E. faecium* ATCC 700221 (**A**,**B**) and *E. faecium* ATCC 700221 treated with 1× MIC of GIE (**C**,**D**) observed by SEM. Magnifications were revealed as (**A**,**B**) = 5000×; (**C**,**D**) = 10,000×.

The morphology of GIE-untreated *E. faecium* ATCC 700221 as the control group revealed a coccus shape and smooth surface (Figure 2A,B). However, the morphology of VREF cells treated with 1× MIC of GIE revealed the damage of bacterial cells (Figure 2C,D; red arrow) compared with the control. The morphological changes included incomplete cell division, damaged cell wall, formation of rough cells, swollen cells, and loss of cellular contents. Moreover, there was disruption of bacterial cells after treatment with GIE.

### 3.5. Antibiofilm Potential of GIE

**Figure 3:**
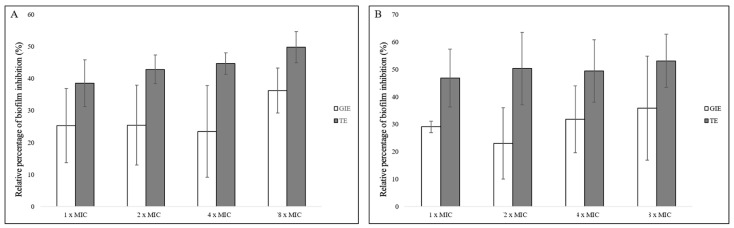
Effects of GIE on the inhibition of biofilm formation (**A**) the inhibition of the established biofilms (**B**) of *E. faecium* ATCC 700221. Medium was used as the negative control. The percent inhibition of each data was compared with its negative control. Data are presented as mean ± standard deviation.

All concentrations of GIE (1, 2, 4, and 8× MIC of GIE) inhibited the biofilm formation ability of *E. faecium* ATCC 700221 compared with the negative control (Figure 3A), although the difference between concentrations was not significant. The concentration of 8× MIC of GIE resulted in the highest inhibition rate of 36.22% ± 7.01%. Moreover, the results revealed a decrease in the viability of mature 2-day-old biofilm-grown cells of *E. faecium* ATCC 700221 after treatment with the 1×, 2×, 4×, and 8× MIC of GIE compared with the negative control (Figure 3B). The highest eradication rate of 35.91% ± 18.93% of the established biofilm of *E. faecium* ATCC 700221 was observed at the 1/8× MIC of GIE.

### 3.6. TPC Content of GIE

The number of total phenolics in GIE was determined using the Folin–Ciocalteu method. The TPC was 0.313 ± 0.01 mgGAE/g plant extract.

### 3.7. DPPH and ABTS Radical Scavenging Activity of GIE

Table 3 shows the results of DPPH and ABTS radical scavenging activity that showed that GIE exhibited an antioxidant property. GIE exhibited strong DPPH radical scavenging activity (76.39% ± 1.90%) that was not significantly different from that of ascorbic acid used as the control (87.20% ± 0.45%) (*p* = 0.673). However, the antioxidant activity of GIE using ABTS radical scavenging activity was significantly lower (17.47% ± 0.75%) than that of ascorbic acid (99.84% ± 0.48%) (*p* = 0.036).

## 4. Discussion

In this study, we focused on the antibacterial and antibiofilm activities of GIE against VREF. *E. faecium* has emerged as a common, high-priority, multi-drug-resistant bacterium. Although commonly found in the gastrointestinal tract, enterococci can cause a variety of infections. They harbor multiple antimicrobial resistance genes and can acquire mutations and/or new resistance genes [1]. As a solution to this problem, there has been a renewed interest in alternative antimicrobial agents such as new antibiotics, bacteriocin, bacteriophage, probiotics, and plant extract [29,30,31].

Our results demonstrated that GIE exerted potential inhibitory effects on the VRE pathogen. GIE showed inhibition zones of 8.33 ± 0.58 mm and 8.67 ± 0.29 mm at 50 and 100 µg/disk concentrations, respectively. Additionally, the MIC and MBC assay results showed that the MBC/MIC ratio was more than four times that considered valuable as a bacteriostatic agent [26,32]. However, to our knowledge, the antimicrobial effects of *G. inodorum* against VREF have not yet been reported, and there are few studies that investigated the antimicrobial activities of *G. Inodorum* [33,34]. Hence, this study was the first to report the antimicrobial activities of GIE against VREF. In a previous study [33], the ethanolic and aqueous extracts of *G. inodorum* (Lour.) Decne. showed no inhibitory effects on *Pseudomonas aeruginosa* ATCC 27853, *Staphylococcus aureus* ATCC 25923, and methicillin-resistant *S. aureus* (MRSA), which was inconsistent with the current study. In another study by Wirasathien (2013) [34], methanol extract of *G. inodorum* showed inhibitory effects against *H. pylori* with MIC 2.5 µg/mL. Additionally, in a previous study in Thailand, GIE showed significant antiparasitic effects against *Plasmodium berghei* parasitemia in mice at 10, 50, and 100 mg/kg doses [16]. However, ethanol is a more efficient solvent for extracting a broader range of phytochemicals. Ethanol is toxic and can pose safety concerns, particularly for therapeutic applications intended for human use. Aqueous extracts are a universal solvent that is non-toxic, readily available, and cost-effective and ensures that the extract is safe for potential therapeutic applications [35]. *G. inodorum* leaves contain various phytochemical constituents, including flavonoids, terpenoids, phenolics, and glycosides [17]. Acetic acid was the major phytochemical presented in the extract, followed by 2-Pyrrolidinone, 1,2,3-Propanetriol, Pyrrolo[1,2-a] pyrazine-1,4-dione, and hexahydro-3-(2-methylpropyl), respectively. It was noticed that Pyrrolo[1,2-a] pyrazine-1,4-dione, hexahydro-3-(2-methylpropyl) was detected at two component RTs that may be its derivative. Acetic acid demonstrates antibacterial properties through various mechanisms such as disruption of bacterial cell membranes, causing leakage of cellular components and subsequent cell death. Its acidic nature lowers the pH environment, perturbing bacterial metabolic processes and vital cellular functions. Acetic acid also inhibits crucial bacterial enzymes, hindering growth and replication. These multifaceted actions highlight acetic acid’s potential as a natural and potent antimicrobial agent [36]. Furthermore, *G. inodorum* exerts antiadipogenesis, antidiabetic, hypoglycemic, and antimalarial effects [37,38]. The phytochemical constituents in *G. inodorum* may contribute to its biological activities, including antimicrobial properties. Similarly, *G. sylvestre*, belonging to the same group as *G. inodorum*, is a potential antimicrobial agent [15]. *G. sylvestre* inhibited the growth of Gram-positive and Gram-negative bacteria, including *S. aureus*, *Bacillus cereus*, *Pseudomonas aeruginosa*, *Escherichia coli*, and *Streptococcus pyogenes* [20,39,40]. The major compounds present in *G. sylvestre* (polyphenols, flavonoids, kaempferol, quinones, anthraquinones, tannins, triterpenoid saponins, and gymnemic acids) contribute to its biological effects.

In this study, the mechanism of action of GIE was investigated by SEM technology. The SEM analysis showed that 1× MIC of GIE disrupted the VREF cells. Additionally, the GIE caused morphological changes that included incomplete cell division, damaged cell wall, formation of rough cells, swollen cells, and loss of cellular contents. These results were in line with those of a previous study by Ngobeni et al. (2020) [41], who showed the disruption of *Bacillus cereus* cell morphology by *Buxus macowanii* medicinal plant. Additionally, in another study, *Lawsonia inermis* plant caused ultrastructure changes in *Streptococcus pneumoniae* [42]. Likewise, *Allium stipitatum* (Persian shallot) caused membrane disruption and several different structure changes in studied Gram-positive and Gram-negative bacteria [25].

Another considerable finding of this study was the antibiofilm properties of GIE. So far, no studies have experimented the antibiofilm properties of GIE against bacteria. Biofilm formation is a protected mode of growth that renders bacterial cells less susceptible to antimicrobials and host immune effector mechanisms, allowing pathogens to thrive in hostile environments [43,44]. Biofilms are inherently up to 1000 times more antibiotic resistant than planktonic bacteria. Therefore, they cause antibiotic resistance in nosocomial settings and antimicrobial treatment failure [44,45]. With their ability to form biofilms, enterococci have become increasingly important opportunistic pathogens across the world. However, antibiofilm drugs are not yet available in clinical settings [46]. This study showed that GIE could inhibit biofilm formation and eradicate mature biofilms of VREF. To date, the antibiofilm effects of various medicinal plants, including *Bergenia ciliata*, *Clematis grata*, *Syzygium gerrardii*, and *Malva sylvestris*, have been reported against the *P. aeruginosa* strain PAO1, *E. faecalis*, and several other bacteria [47,48,49]. The biofilm inhibition by plant extracts may be due to their interference with forces (electrostatic interactions, van der Waals, Brownian, and sedimentation forces) that favor bacteria to adhere to surfaces [48]. There is also the possibility that the plant extracts inhibit the availability of nutrients that are crucial for bacterial growth and adhesion [48].

Bacterial infections often trigger an immune response that, while essential for fighting pathogens, can lead to the production of free radicals. These reactive oxygen species (ROS) can cause significant damage to cellular structures, DNA, and proteins, exacerbating the infection and leading to further complications. Antioxidants mitigate this damage by neutralizing these radicals, thereby playing a crucial role in both managing the oxidative stress and supporting the body’s recovery. This dual action highlights the potential of antioxidant treatments in enhancing outcomes in bacterial infections [18]. In this study, we found that GIE contained phenolic compounds (TPC = 0.313 ± 0.01 mgGAE/g) and exhibited antioxidant activities using DPPH (76.39% ± 1.90%) and ABTS (17.47% ± 0.75%) radical scavenging activities. These findings were consistent with those observed by Chanwitheesuk et al. (2005) [50], who found the highest antioxidant activity in *G. inodorum*. Another study showed that the phenolic and flavonoid contents of *G. inodorum* were 0.81 ± 0.01 mgGAE/g and 4.99 ± 0.63 mgCE/g of dry weight, respectively [37]. The antioxidant and anti-inflammatory properties of *G. Inodorum* were also reported by Dunkhunthod et al. (2021) [28] in Thailand. The high antioxidant capacity could be partially attributed to the formation of membrane structures that exhibit resistance to detergent solubilization. In these structures, phospholipids have tightly packed acyl chains and highly hydrated phosphate groups. Certain compounds have been found to be crucial for promoting carboxyfluorescein leakage from bacterial model membranes by galloylated catechins, which indicates their antibacterial activity [51].

This study had limitation as follows: the lack of determination of active phytoconstituents of GIE, the lack of experiment of GIE on other bacteria including Gram-negative species or bacteria from clinical origin, and the lack of the in vivo assay. The detection of the compounds in the plant extract should be further investigated by Liquid Chromatography–Mass Spectrometry (LCMS) analysis.

## 5. Conclusions

The findings of this study indicate that aqueous crude extract of *G. inodorum* possesses antimicrobial activity against vancomycin-resistant *E. faecium* ATCC 700221, demonstrating both antibacterial and antibiofilm effects. The extract showed an inhibitory effect on biofilm formation and was able to eradicate mature biofilms. However, the observed antimicrobial activity is not very high, suggesting that GIE alone may not be sufficient to treat enterococcal infections effectively. These effects of GIE on VREF were reported for the first time. Moreover, GIE contained phenols and exhibited antioxidant activities. pH measurements were not carried out during the preparation and testing of the extracts. Therefore, future studies should focus on identifying the active phytoconstituents, optimizing extraction methods, and validating the findings through in vivo assays and broader bacterial spectrum testing.

## Figures and Tables

**Table 1:** Phytochemical composition of *Gymnema inodorum* extract.

Component RT	Percentage of Total (%)	Formula	Compound Name
6.2696	2.3	C_8_H_17_N	1-Propanamine, 2-methyl-N-(2-methylpropylidene)-
7.8194	2.31	C_9_H_19_N	2-Butyl-(2-methylbutylidene)-amine
17.2102	2.37	C_3_H_6_O_2_	2-Propanone, 1-hydroxy-
18.1513	0.5	C_6_H_8_N_2_	Pyrazine, 2,5-dimethyl-
23.3098	12.8	C_2_H_4_O_2_	Acetic acid
26.8357	2.55	C_3_H_6_O_2_	Propanoic acid
30.3095	1.04	C_4_H_6_O_2_	2(3H)-Furanone, dihydro-
31.5388	0.95	C_11_H_18_N_2_	Pyrazine, 2,5-dimethyl-3-(3-methylbutyl)-
31.8858	0.86	C_5_H_10_O_2_	Pentanoic acid
43.4793	0.62	C_6_H_6_O	Phenol
44.3675	0.54	C_6_H_8_O_3_	Hydroxy dimethyl furanone
44.5557	9.35	C_4_H_7_NO	2-Pyrrolidinone
48.6026	1.04	C_4_H_6_O_3_	2-Hydroxy-gamma-butyrolactone
49.3853	2.31	C_9_H_10_O_2_	2-Methoxy-4-vinylphenol
51.3848	1.33	C_6_H_8_O_4_	4H-Pyran-4-one, 2,3-dihydro-3,5-dihydroxy-6-methyl-
52.6847	6.89	C_3_H_8_O_3_	1,2,3-Propanetriol
55.0081	5.9	C_8_H_8_O	Benzofuran, 2,3-dihydro-
55.7081	0.84	C_5_H_5_NO	3-Pyridinol
61.1372	1.45	C_20_H_40_O	Phytol
64.2782	1.76	C_11_H_11_N	3-Methyl-4-phenyl-1H-pyrrole
68.1604	3.83	C_16_H_32_O_2_	n-Hexadecanoic acid
71.5484	2.08	C_10_H_16_N_2_O_2_	Cyclo(L-prolyl-L-valine)
73.4013	0.8	C_18_H_36_O_2_	Octadecanoic acid
74.2189	4.64	C_11_H_18_N_2_O_2_	Pyrrolo[1,2-a] pyrazine-1,4-dione, hexahydro-3-(2-methylpropyl)-
75.8247	4.4	C_11_H_18_N_2_O_2_	Pyrrolo[1,2-a] pyrazine-1,4-dione, hexahydro-3-(2-methylpropyl)-
78.5245	2.42	C_18_H_30_O_2_	9,12,15-Octadecatrienoic acid, (Z,Z,Z)-

**Table 2:** Antimicrobial activity of GIE against *E. faecium* ATCC 700221.

	Zone of Inhibition (mm)	
GIE (50 µg/disk)	8.33 ± 0.58	
GIE (100 µg/disk)	8.67 ± 0.29	
DW	-	
Tetracycline	25 ± 0.00	
	MIC (mg/mL)	MBC (mg/mL)
GIE	125	≥250
Tetracycline	≤0.025	≤0.025

GIE; aqueous crude extract of *G. inodorum*. Values are mean ± standard deviation (*n* = 3) and represent three independent experiments with similar results.

**Table 3:** The DPPH and ABTS^+^ scavenging activities of GIE and standard compounds.

Sample	DPPH Radical Scavenging Activity (%)	ABTS^+^ Radical Scavenging Activity (%)
GIE	76.39 ± 1.90	17.47 ± 0.75 ^a^
Ascorbic acid	87.20 ± 0.45	99.84 ± 0.48 ^b^

GIE; aqueous crude extract of *G. inodorum*. Values are mean ± standard deviation (*n* = 3) and represent three independent experiments with similar outcomes. Different letters indicate statistically significant differences between sample groups, with *p* < 0.05 considered statistically significant.

## Data Availability

The original contributions presented in the study are included in the article/supplementary material, further inquiries can be directed to the corresponding author.

## Supplementary Materials

The following supporting information can be downloaded at https://www.mdpi.com/article/10.3390/microorganisms12071399/s1, Figure S1. GC–MS spectrum of 2-Pyrrolidinone; Figure S2. GC–MS spectrum of 1,2,3-Propanetriol; Figure S3. GC–MS spectrum of Benzofuran, 2,3-dihydro; Figure S4. GC–MS spectrum of n-Hexadecanoic acid; Figure S5. GC–MS spectrum of Pyrrolo[1,2-a] pyrazine-1,4-dione, hexahydro-3-(2-methylpropyl); Figure S6. Determination of minimum inhibitory concentration (MIC) and minimum bactericidal concentration (MBC) of GIE against *E. faecium* ATCC 700221; Table S1. Antibiogram of *E. faecium* ATCC 700221.

## References

[1] Joshi S., Shallal A., Zervos M. (2021). Vancomycin-Resistant Enterococci: Epidemiology, Infection Prevention, and Control. Infect. Dis. Clin. N. Am..

[2] Gao W., Howden B.P., Stinear T.P. (2018). Evolution of virulence in *Enterococcus faecium*, a hospital-adapted opportunistic pathogen. Curr. Opin. Microbiol..

[3] Schultz F., Anywar G., Tang H., Chassagne F., Lyles J.T., Garbe L.-A., Quave C.L. (2020). Targeting ESKAPE pathogens with anti-infective medicinal plants from the Greater Mpigi region in Uganda. Sci. Rep..

[4] van Hal S.J., Willems R.J.L., Gouliouris T., Ballard S.A., Coque T.M., Hammerum A.M., Hegstad K., Westh H.T., Howden B.P., Malhotra-Kumar S. (2021). The global dissemination of hospital clones of *Enterococcus faecium*. Genome Med..

[5] Lam P.W., Kozak R.A., Eshaghi A., Avaness M., Salt N., Patel S.N., Simor A.E., Leis J.A. (2018). Nosocomial outbreak of vanD-carrying vancomycin-resistant *Enterococcus faecium*. Infect. Control Hosp. Epidemiol..

[6] dos Santos L.D.R., Furlan J.P.R., Gallo I.F.L., Ramos M.S., Savazzi E.A., Stehling E.G. (2021). Occurrence of multidrug-resistant *Enterococcus faecium* isolated from environmental samples. Lett. Appl. Microbiol..

[7] Sattari-Maraji A., Jabalameli F., Node Farahani N., Beigverdi R., Emaneini M. (2019). Antimicrobial resistance pattern, virulence determinants and molecular analysis of *Enterococcus faecium* isolated from children infections in Iran. BMC Microbiol..

[8] Wei Y., Palacios Araya D., Palmer K.L. (2024). *Enterococcus faecium*: Evolution, adaptation, pathogenesis and emerging therapeutics. Nat. Rev. Microbiol..

[9] Barbosa-Ribeiro M., Gomes B., Arruda-Vasconcelos R., Monteiro I.A., Costa M.J.F., Sette-de-Souza P.H. (2024). Antibiotic Resistance Profile of Clinical Strains of Enterococci from Secondary/Persistent Endodontic Infections: What do We Know? A Systematic Review of Clinical Studies. J. Endod..

[10] Saeloh D., Visutthi M. (2021). Efficacy of Thai Plant Extracts for Antibacterial and Anti-Biofilm Activities against Pathogenic Bacteria. Antibiotics.

[11] Kohno J., Kawamura T., Kikuchi A., Akaishi T., Takayama S., Ishii T. (2021). A Japanese traditional medicine Hochuekkito promotes negative conversion of vancomycin-resistant Enterococci. Sci. Rep..

[12] Nowak A., Cybulska K., Makuch E., Kucharski Ł., Różewicka-Czabańska M., Prowans P., Czapla N., Bargiel P., Petriczko J., Klimowicz A. (2021). In Vitro Human Skin Penetration, Antioxidant and Antimicrobial Activity of Ethanol-Water Extract of Fireweed (*Epilobium angustifolium* L.). Molecules.

[13] Satdive R.K., Abhilash P., Fulzele D.P. (2003). Antimicrobial activity of *Gymnema sylvestre* leaf extract. Fitoterapia.

[14] Tajuddeen N., Sani Sallau M., Muhammad Musa A., James Habila D., Muhammad Yahaya S. (2014). Flavonoids with antimicrobial activity from the stem bark of *Commiphora pedunculata* (Kotschy & Peyr. ) Engl. Nat. Prod. Res..

[15] Kahksha, Alam O., Naaz S., Sharma V., Manaithiya A., Khan J., Alam A. (2022). Recent developments made in the assessment of the antidiabetic potential of gymnema species—From 2016 to 2020. J. Ethnopharmacol..

[16] Ounjaijean S., Romyasamit C., Somsak V. (2021). Evaluation of Antimalarial Potential of Aqueous Crude *Gymnema Inodorum* Leaf Extract against *Plasmodium berghei* Infection in Mice. Evid. Based Complement. Alternat. Med..

[17] Jeytawan N., Yadoung S., Jeeno P., Yana P., Sutan K., Naksen W., Wongkaew M., Sommano S.R., Hongsibsong S. (2022). Antioxidant and Phytochemical Potential of and Phytochemicals in *Gymnema inodorum* (Lour.) Decne in Northern Thailand. Plants.

[18] Mittal M., Siddiqui M.R., Tran K., Reddy S.P., Malik A.B. (2014). Reactive oxygen species in inflammation and tissue injury. Antioxid. Redox Signal..

[19] Pham-Huy L.A., He H., Pham-Huy C. (2008). Free radicals, antioxidants in disease and health. Int. J. Biomed. Sci..

[20] Arun L.B., Arunachalam A.M., Arunachalam K.D., Annamalai S.K., Kumar K.A. (2014). In vivo anti-ulcer, anti-stress, anti-allergic, and functional properties of gymnemic acid isolated from *Gymnema sylvestre* R Br. BMC Complement. Altern. Med..

[21] Nunta R., Khemacheewakul J., Sommanee S., Mahakuntha C., Chompoo M., Phimolsiripol Y., Jantanasakulwong K., Kumar A., Leksawasdi N. (2023). Extraction of gymnemic acid from *Gymnema inodorum* (Lour.) Decne. leaves and production of dry powder extract using maltodextrin. Sci. Rep..

[22] Boonyapranai K., Surinkaew S., Somsak V., Rattanatham R. (2021). Protective Effects of *Gymnema inodorum* Leaf Extract on Plasmodium berghei-Induced Hypoglycemia, Dyslipidemia, Liver Damage, and Acute Kidney Injury in Experimental Mice. J. Parasitol. Res..

[23] Ounjaijean S., Sukati S., Somsak V., Sarakul O. (2021). The Potential Role of *Gymnema inodorum* Leaf Extract Treatment in Hematological Parameters in Mice Infected with *Plasmodium berghei*. J. Trop. Med..

[24] (2021). Performance Standards for Antimicrobial Susceptibility Testing.

[25] Kulnanan P., Chuprom J., Thomrongsuwannakij T., Romyasamit C., Sangkanu S., Manin N., Nissapatorn V., Pereira M.d.L., Wilairatana P., Kitpipit W. (2021). Antibacterial, antibiofilm, and anti-adhesion activities of Piper betle leaf extract against Avian pathogenic *Escherichia coli*. Arch. Microbiol..

[26] Sornsenee P., Chatatikun M., Mitsuwan W., Kongpol K., Kooltheat N., Sohbenalee S., Pruksaphanrat S., Mudpan A., Romyasamit C. (2021). Lyophilized cell-free supernatants of Lactobacillus isolates exhibited antibiofilm, antioxidant, and reduces nitric oxide activity in lipopolysaccharide-stimulated RAW 264.7 cells. PeerJ..

[27] Zongo C., Savadogo A., Ouattara L., Bassole I., Ouattara C., Ouattara A., Barro N., Koudou J., Traore A. (2010). Polyphenols content, antioxidant and antimicrobial activities of *Ampelocissus grantii* (Baker) Planch. (Vitaceae): A medicinal plant from Burkina Faso. Int. J. Pharmacol..

[28] Dunkhunthod B., Talabnin C., Murphy M., Thumanu K., Sittisart P., Eumkeb G. (2021). *Gymnema inodorum* (Lour.) Decne. Extract Alleviates Oxidative Stress and Inflammatory Mediators Produced by RAW264.7 Macrophages. Oxid. Med. Cell. Longev..

[29] Sato M., Tanaka H., Oh-Uchi T., Fukai T., Etoh H., Yamaguchi R. (2004). Antibacterial activity of phytochemicals isolated from *Erythrina zeyheri* against vancomycin-resistant enterococci and their combinations with vancomycin. Phytother. Res..

[30] Miller W.R., Murray B.E., Rice L.B., Arias C.A. (2016). Vancomycin-Resistant Enterococci: Therapeutic Challenges in the 21st Century. Infect. Dis. Clin. N. Am..

[31] Plupjeen S.N., Chawjiraphan W., Charoensiddhi S., Nitisinprasert S., Nakphaichit M. (2020). *Lactococcus lactis* KA-FF 1-4 reduces vancomycin-resistant enterococci and impacts the human gut microbiome. 3 Biotech..

[32] Levison M.E. (2004). Pharmacodynamics of antimicrobial drugs. Infect. Dis. Clin. N. Am..

[33] Janta K., Thaharn W. (2018). Antibacterial activity of medicinal plant extracts against some pathogenic bacteria causing skin diseases. Prog. Appl. Sci. Technol..

[34] Wirasathien L. (2013). Anti-*Helicobacter pylori* activity of local edible plants. Planta Medica.

[35] Plaskova A., Mlcek J. (2023). New insights of the application of water or ethanol-water plant extract rich in active compounds in food. Front Nutr..

[36] Pangprasit N., Srithanasuwan A., Suriyasathaporn W., Pikulkaew S., Bernard J.K., Chaisri W. (2020). Antibacterial Activities of Acetic Acid against Major and Minor Pathogens Isolated from Mastitis in Dairy Cows. Pathogens.

[37] Tiamyom K., Sirichaiwetchakoon K., Hengpratom T., Kupittayanant S., Srisawat R., Thaeomor A., Eumkeb G. (2019). The Effects of *Cordyceps sinensis* (Berk.) Sacc. and *Gymnema inodorum* (Lour.) Decne. Extracts on Adipogenesis and Lipase Activity In Vitro. Evid. Based Complement. Altern. Med..

[38] Nuchuchua O., Inpan R., Srinuanchai W., Karinchai J., Pitchakarn P., Wongnoppavich A., Imsumran A. (2023). Phytosome Supplements for Delivering *Gymnema inodorum* Phytonutrients to Prevent Inflammation in Macrophages and Insulin Resistance in Adipocytes. Foods.

[39] Ramalingam R., Dhand C., Leung C.M., Ong S.T., Annamalai S.K., Kamruddin M., Verma N.K., Ramakrishna S., Lakshminarayanan R., Arunachalam K.D. (2019). Antimicrobial properties and biocompatibility of electrospun poly-ε-caprolactone fibrous mats containing *Gymnema sylvestre* leaf extract. Mater. Sci. Eng. C.

[40] Gomathi M., Prakasam A., Rajkumar P.V., Rajeshkumar S., Chandrasekaran R., Anbarasan P.M. (2020). Green synthesis of silver nanoparticles using *Gymnema sylvestre* leaf extract and evaluation of its antibacterial activity. South Afr. J. Chem. Eng..

[41] Ngobeni B., Mashele S.S., Malebo N.J., van der Watt E., Manduna I.T. (2020). Disruption of microbial cell morphology by *Buxus macowanii*. BMC Complement. Med. Ther..

[42] Tafroji W., Margyaningsih N.I., Khoeri M.M., Paramaiswari W.T., Winarti Y., Salsabila K., Putri H.F.M., Siregar N.C., Soebandrio A., Safari D. (2022). Antibacterial activity of medicinal plants in Indonesia on *Streptococcus pneumoniae*. PLoS ONE.

[43] Del Pozo J.L. (2018). Biofilm-related disease. Expert Rev. Anti Infect. Ther..

[44] Xiong Y., Chen J., Sun X., Xu G., Li P., Deng Q., Yu Z., Chen Z., Zheng J. (2020). The Antibacterial and Antibiofilm Activity of Telithromycin Against *Enterococcus spp*. Isolated From Patients in China. Front. Microbiol..

[45] Tolker-Nielsen T. (2015). Biofilm Development. Microbiol Spectr..

[46] Zeng X., She P., Zhou L., Li S., Hussain Z., Chen L., Wu Y. (2021). Drug repurposing: Antimicrobial and antibiofilm effects of penfluridol against *Enterococcus faecalis*. Microbiologyopen.

[47] Alam K., Al Farraj D.A., Mah-E-Fatima S., Yameen M.A., Elshikh M.S., Alkufeidy R.M., Mustafa A.E.-Z.M., Bhasme P., Alshammari M.K., Alkubaisi N.A. (2020). Anti-biofilm activity of plant derived extracts against infectious pathogen-*Pseudomonas aeruginosa* PAO1. J. Infect. Public Health.

[48] Famuyide I.M., Aro A.O., Fasina F.O., Eloff J.N., McGaw L.J. (2019). Antibacterial and antibiofilm activity of acetone leaf extracts of nine under-investigated south African Eugenia and Syzygium (Myrtaceae) species and their selectivity indices. BMC Complement. Altern. Med..

[49] Fathi M., Ghane M., Pishkar L. (2021). Phytochemical Composition, Antibacterial, and Antibiofilm Activity of *Malva sylvestris* Against Human Pathogenic Bacteria. Jundishapur J. Nat. Pharm. Prod..

[50] Chanwitheesuk A., Teerawutgulrag A., Rakariyatham N. (2005). Screening of antioxidant activity and antioxidant compounds of some edible plants of Thailand. Food Chem..

[51] Caturla N., Vera-Samper E., Villalaín J., Mateo C.R., Micol V. (2003). The relationship between the antioxidant and the antibacterial properties of galloylated catechins and the structure of phospholipid model membranes. Free Radic. Biol. Med..

