# Supplementary material for: Antibacterial, Antibiofilm, and Antioxidant Activities of Aqueous Crude Gymnema inodorum Leaf Extract against Vancomycin-Resistant Enterococcus faecium
Source: Microorganisms. 2024 Jul 11;12(7):1399. doi: 10.3390/microorganisms12071399 (PMC11278954; doi:10.3390/microorganisms12071399)
Supplement: Supplementary file 1 [file microorganisms-12-01399-s001.zip › microorganisms-3095449-supplementary.pdf]

Supplementary data

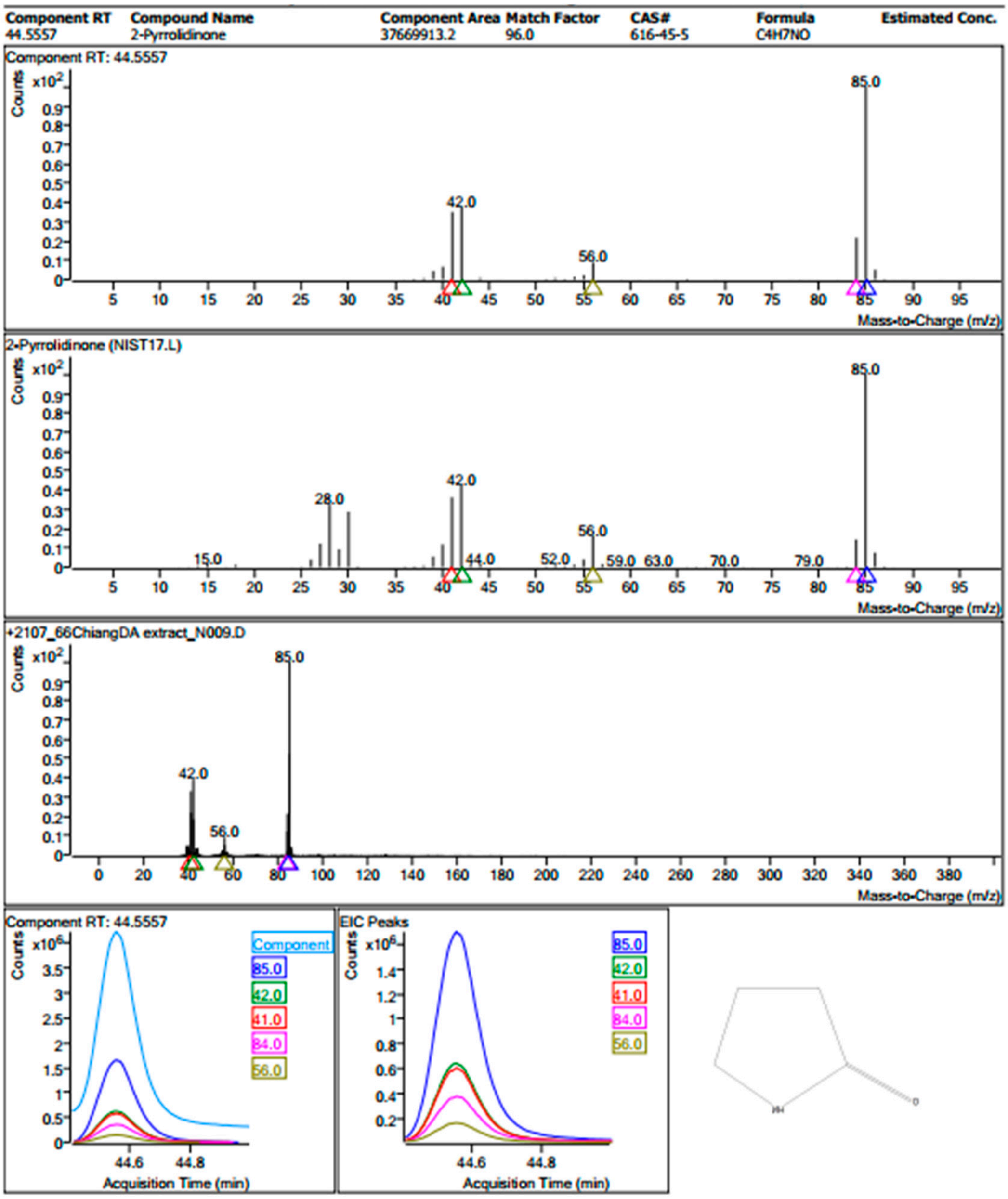

Figure S1. GC-MS spectrum of 2-Pyrrolidinone.

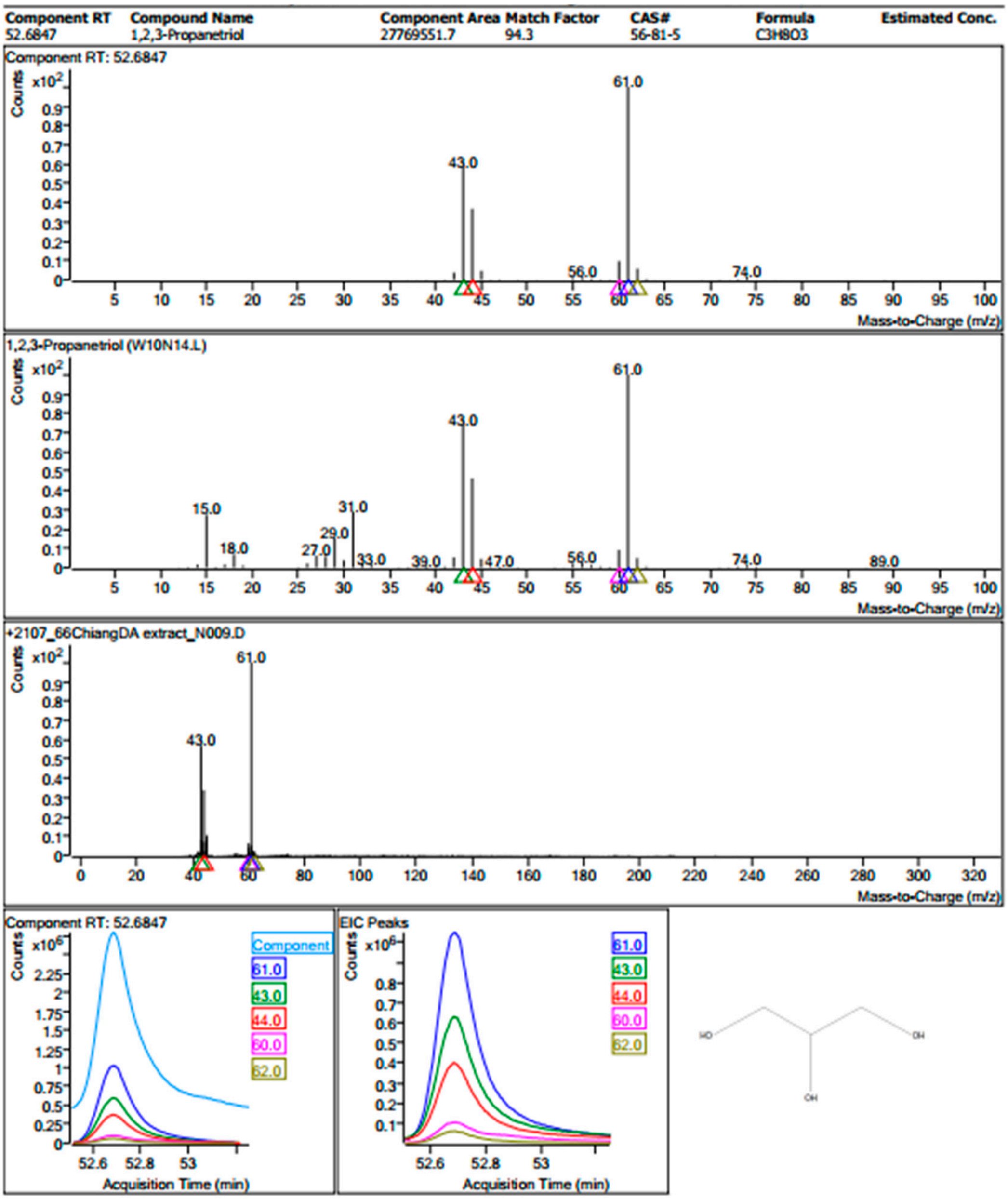

Figure S2. GC-MS spectrum of 1,2,3-Propanetriol.

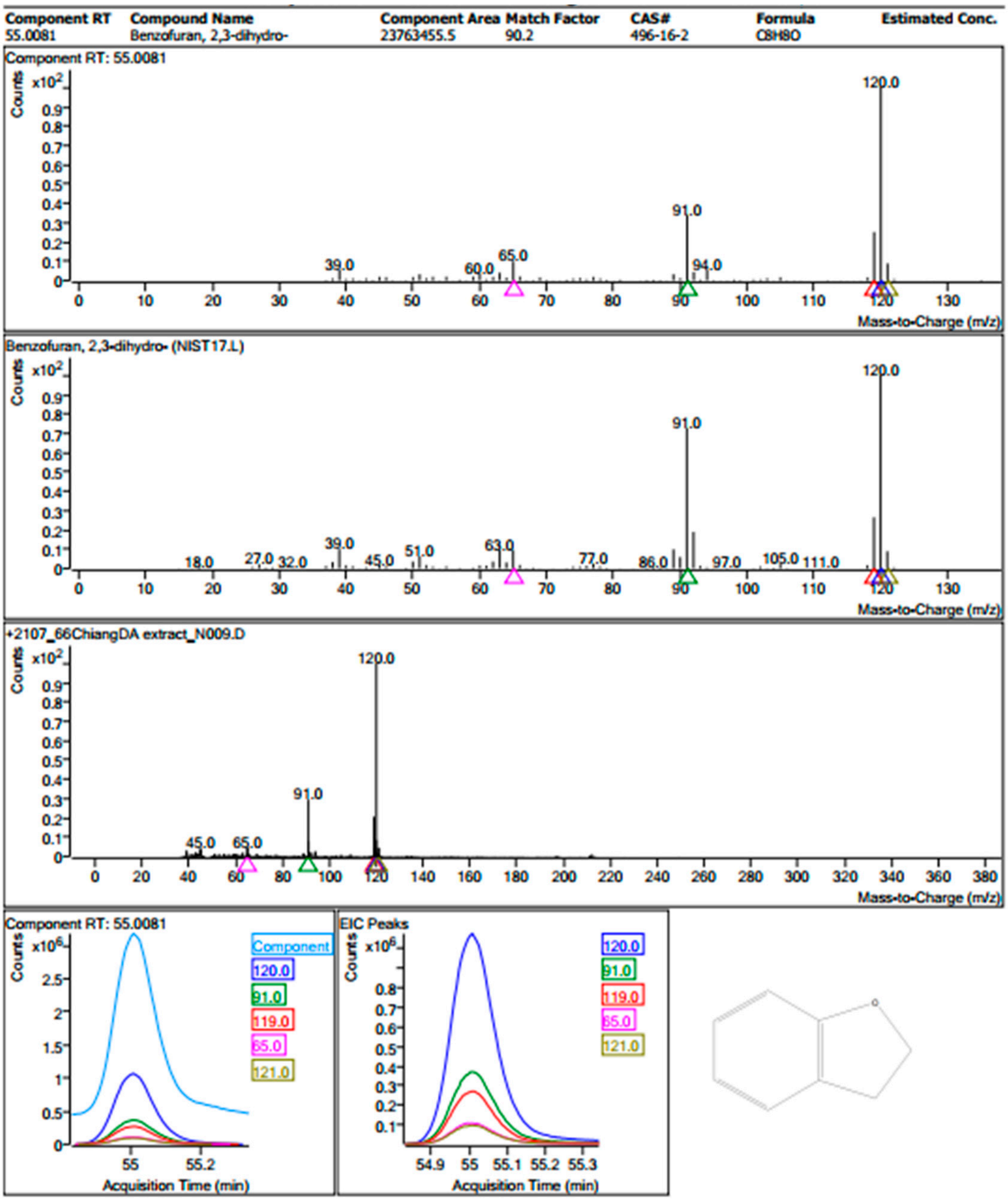

Figure S3. GC-MS spectrum of Benzofuran, 2,3-dihydro.

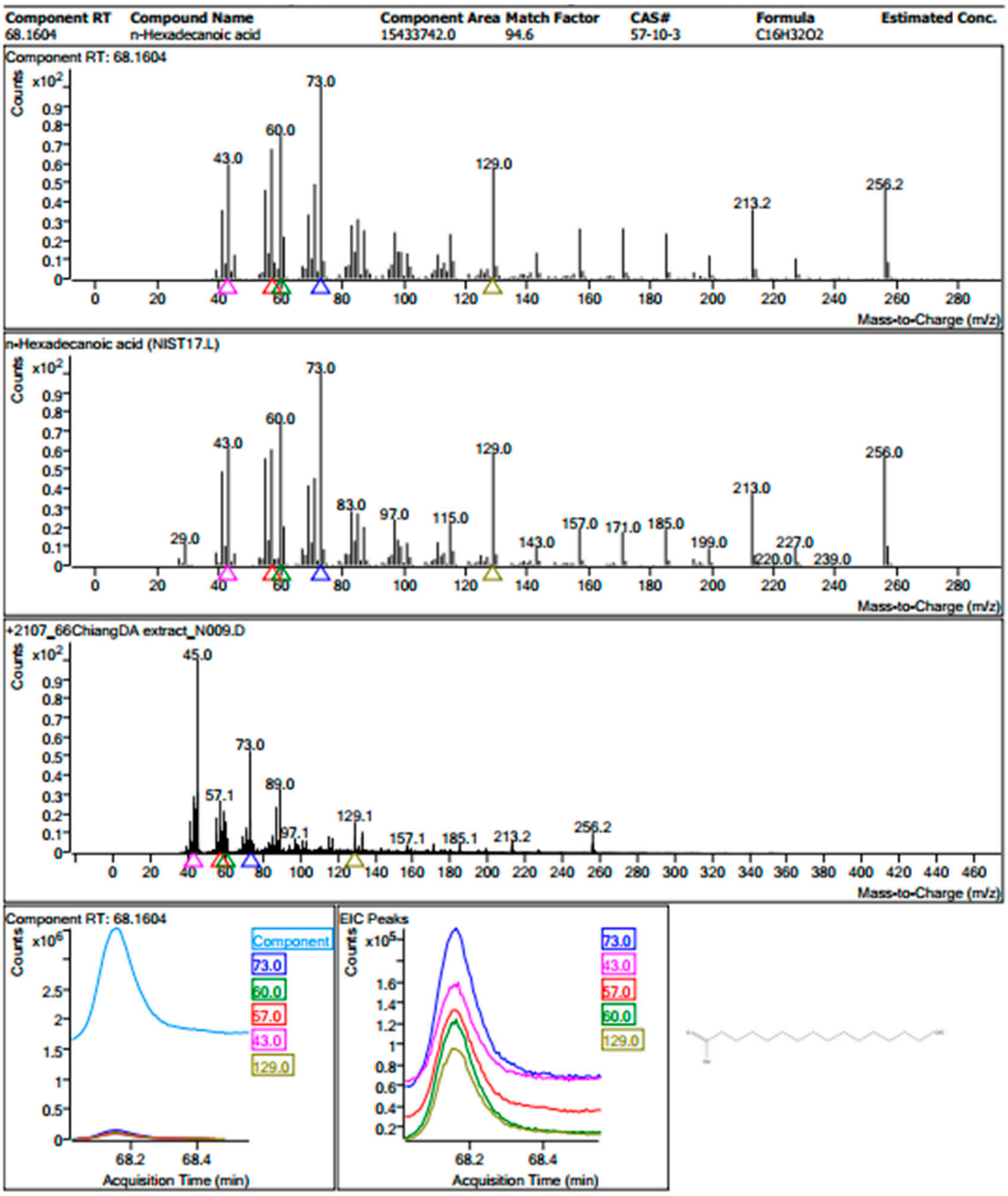

Figure S4. GC-MS spectrum of n-Hexadecanoic acid.

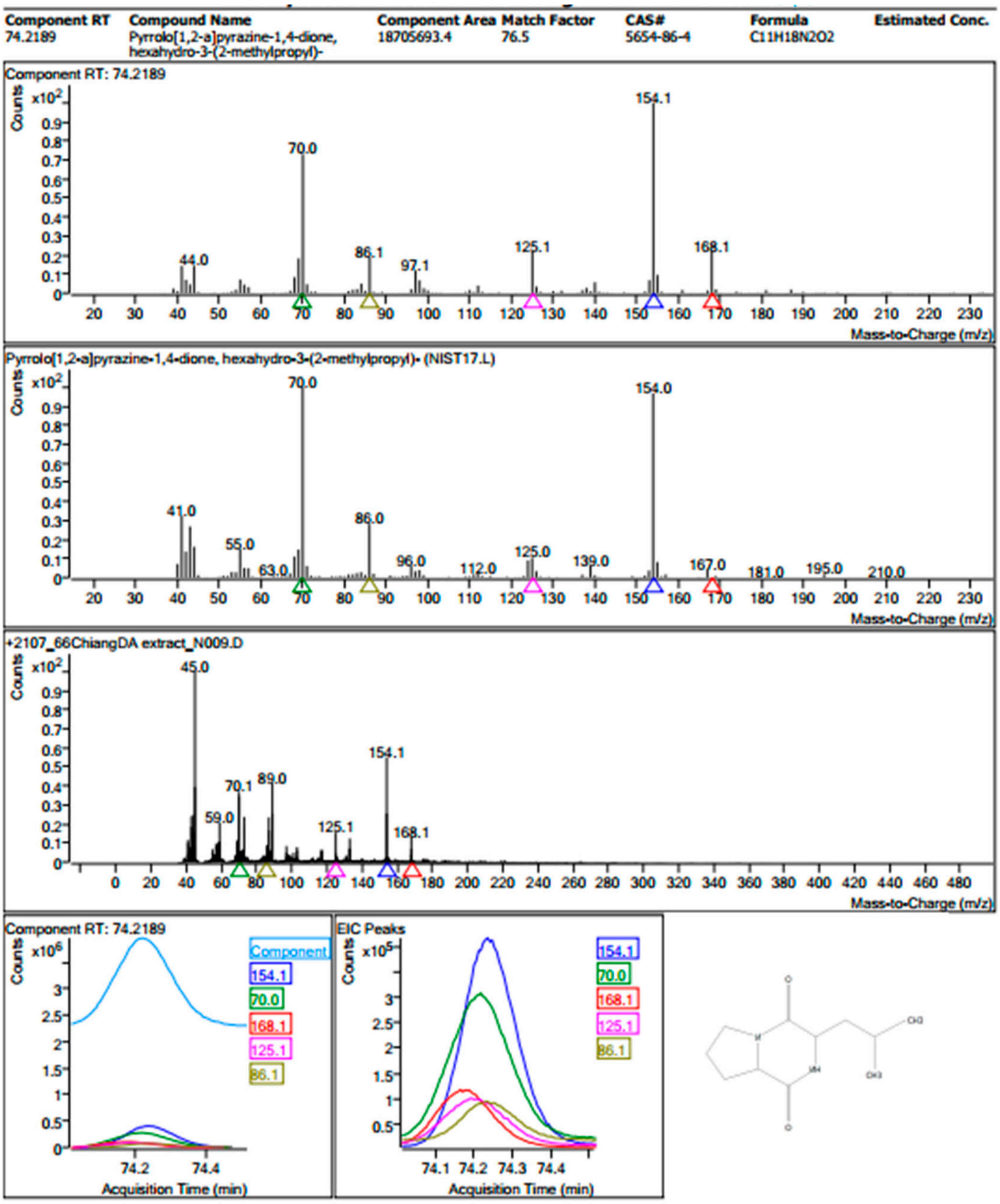

Figure S5. GC-MS spectrum of Pyrrolo[1,2-a] pyrazine-1,4-dione, hexahydro-3-(2-methylpropyl).

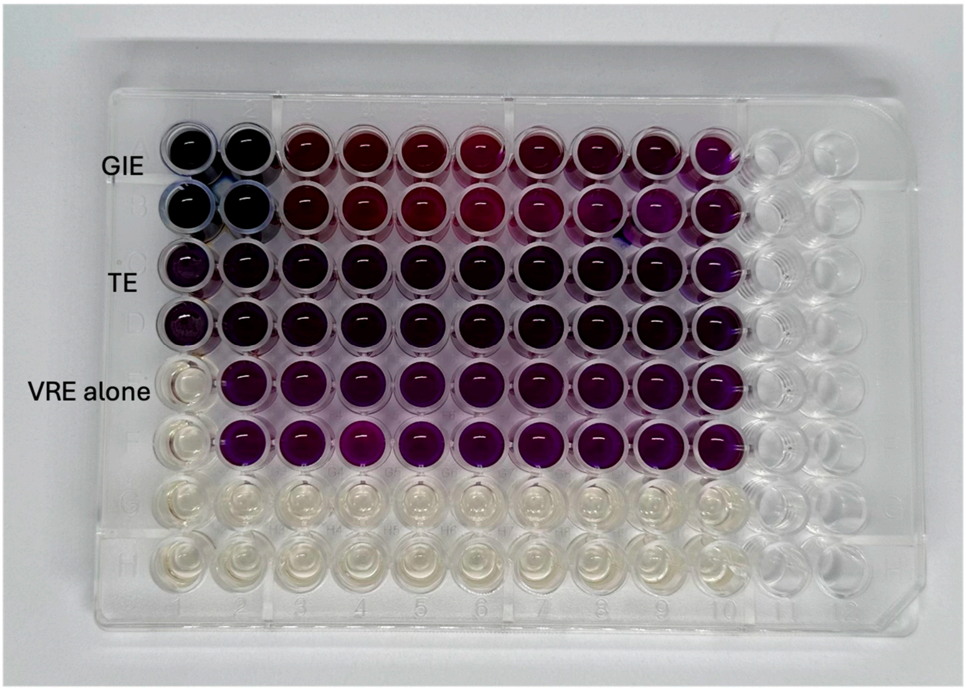

Figure S6. Determination of minimum inhibitory concentration (MIC) and minimum bactericidal concentration (MBC) of GIE against *E. faecium* ATCC 700221

Table S1. Antibiogram of *E. faecium* ATCC 700221

Isolates	Ampicillin	Tetracycline	Erythromycin	Clidamycim	Vancomycin
E. faecium ATCC 700221	S	S	R	R	R
